# A lever-like transduction pathway for long-distance chemical- and mechano-gating of the mechanosensitive Piezo1 channel

**DOI:** 10.1038/s41467-018-03570-9

**Published:** 2018-04-03

**Authors:** Yanfeng Wang, Shaopeng Chi, Huifang Guo, Guang Li, Li Wang, Qiancheng Zhao, Yu Rao, Liansuo Zu, Wei He, Bailong Xiao

**Affiliations:** 1 School of Pharmaceutical Sciences, Tsinghua University, Beijing, 100084 China; 2 Tsinghua-Peking Joint Center for Life Sciences, Tsinghua University, Beijing, 100084 China; 3 IDG/McGovern Institute for Brain Research, Tsinghua University, Beijing, 100084 China; 4 Institute of Medicinal Biotechnology, Chinese Academy of Medical Sciences, Peking Union Medical College, Beijing, 100050 China

## Abstract

Piezo1 represents a prototype of eukaryotic mechanotransduction channels. The full-length 2547-residue mouse Piezo1 possesses a unique 38-transmembrane-helix (TM) topology and is organized into a three-bladed, propeller-shaped architecture, comprising a central ion-conducting pore, three peripheral blade-like structures, and three 90-Å-long intracellular beam-resembling structures that bridge the blades to the pore. However, how mechanical force and chemicals activate the gigantic Piezo1 machinery remains elusive. Here we identify a novel set of Piezo1 chemical activators, termed Jedi, which activates Piezo1 through the extracellular side of the blade instead of the C-terminal extracellular domain of the pore, indicating long-range allosteric gating. Remarkably, Jedi-induced activation of Piezo1 requires the key mechanotransduction components, including the two extracellular loops in the distal blade and the two leucine residues in the proximal end of the beam. Thus, Piezo1 employs the peripheral blade-beam-constituted lever-like apparatus as a designated transduction pathway for long-distance mechano- and chemical-gating of the pore.

## Abstract (web summary)

Piezo1 is a large trimeric ion channel activated by mechanical stimulus. Here the authors identify chemical activators of Piezo1 that utilize a lever-like mechanotransduction pathway for long-range allosteric gating.

## Introduction

Mechanosensitive (MS) ion channels are molecular force transducers that specialize in rapidly converting various mechanical stimuli into electrochemical signals for controlling key biological activities such as touch, vascular development, and blood pressure regulation^1–3^. It is thus imperative to understand how this process, termed mechanogating, precisely occurs. While significant progresses have been made in studying prokaryotic MS channels (i.e., the MS channel of large conductance)^4^, we know relatively little about the mechanogating mechanisms of mammalian MS cation channels.

The evolutionarily conserved Piezo proteins, including Piezo1 and Piezo2, have been established as the long-sought-after mammalian MS cation channels^5–8^. In mice, Piezos play critical roles in various mechanotransduction processes^3^. For instance, Piezo1 is critically involved in vascular development, arterial remodeling and blood pressure regulation^9–13^, neural stem cell fate determination^14^, axon guidance and growth^15^, neuron-astrocyte interactions^16^, and endothelium homeostasis^17,18^, while Piezo2 functions as a major mechanotransduction channel in sensing gentle touch^19–22^, proprioception^23^, airway stretch, and lung inflation^24^. In humans, mutations of *Piezo* genes resulting in altered channel functions have been linked to a number of genetic diseases involving mechanotransduction^25–31^. These studies demonstrate the functional importance of Piezo channels and their potential as therapeutic targets.

Mouse Piezo1 (mPiezo1) is characteristically activated by various forms of mechanical stimulation, including poking, stretching, shear stress, and substrate deflection^5,32–35^. Importantly, purified mPiezo1 proteins form MS cation channels when reconstituted into lipid bilayers^6,36^. Thus, Piezo1 represents a prototype for understanding the mechanogating and ion-permeating mechanisms of mammalian MS cation channels, which will lead to a better understanding of their biological roles in health and disease.

**Fig. 1:**
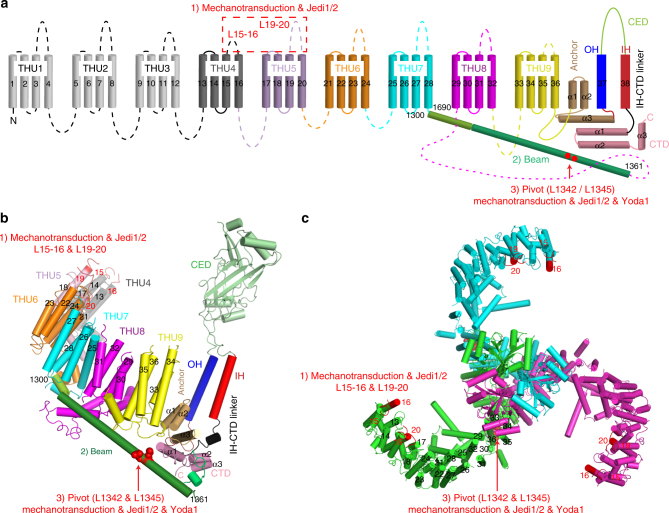
Topological and structural illustration of the designated mechanical and chemical activation of Piezo1. **a**, **b** A topological model (**a**) and structural model (based on the PDB 5Z10) (**b**) of Piezo1 showing the featured domains revealed from the reported cryo-EM structure of Piezo1^39^, including the nine repetitive THUs, beam, anchor, and the C-terminal pore module comprising the OH, CED, IH, and CTD. The N-terminal THU1-THU3 were not structurally resolved. **c** Top view of the trimeric Piezo1 channel showing the three-bladed, propeller-like architecture and location of the L15-16 and L19-20 at the distal end of the blade. Key structural components involved in mechanical and chemical activation of Piezo1, including the extracellular loops of L15-16 and L19-20, beam, and L1342/L1345 (proposed to form the pivot of the beam), are labeled in **a**–**c**. As illustrated in steps (1)–(3), the blade-beam might form a designated transduction pathway for propagating action from the extracellular side of the distal blade to the central pore via utilizing the intracellular beam as a lever-like apparatus

The full-length mPiezo1 has 2547 amino acids in each monomer and bears no sequence homology with other known ion channels^5^. The cryo-electron microscopy structure of mPiezo1 reveals that it trimerizes to form a unique three-bladed, propeller-shaped architecture, comprising a central ion-conducting pore module, three peripheral blades, and three 90 Å-long intracellular beams^7^ (Fig. 1). The central pore, which is encoded by the C-terminal ~360 residues (residues 2189–2547) containing the last two transmembrane-helices (TMs; [outer helix (OH) and inner helix]; Fig. 1a, b), determines the fundamental pore properties, including unitary conductance, ion selectivity, and pore blockage^8,37,38^. The rest of the protein forms the peripheral blade and beam, which define the unique architecture of the Piezo1 channel^39–41^ (Fig. 1). Remarkably, the highly curved blade is composed of 36 TMs, which are folded into nine repetitive transmembrane helical units (THUs) consisting of 4 TMs each^39^. The beam is formed by residues H1300-S1362, and connects the peripheral THUs to the pore via the interfaces of the C-terminal domain, anchor-resembling domain and OH (Fig. 1). Combining structural and functional characterizations, we have identified that the two extracellular loops of TM15-16 (L15-16) and TM19-20 (L19-20) in the distal blade and the two leucine residues L1342 and L1345 in the proximal end of the beam play critical roles in the mechanical activation of Piezo1^39^ (Fig. 1). However, whether these remotely located mechanotransduction components constitute a designated mechanogating pathway remains unclear. Furthermore, how other forms of stimuli such as chemicals activate Piezo channels is unknown.

Here we have first conducted a high-throughput screening to identify two novel chemical activators of Piezo1. Given that the previously reported Piezo1 chemical activator, Yoda1^42^, is named after a Jedi Knight, we term Piezo1 chemical activators in general as Jedi (i.e., Jedi1/2 for the Piezo1 activators identified in this study). We have next found that Jedi1/2 utilize the key mechanotransduction components to activate Piezo1. Our studies suggest that Piezo1 employ the peripheral blade-beam structure as a designated transduction pathway for long-distance mechano- and chemical-gating of the central ion-conducting pore.

## Results

### Identification of novel Piezo1 chemical activators

**Fig. 2:**
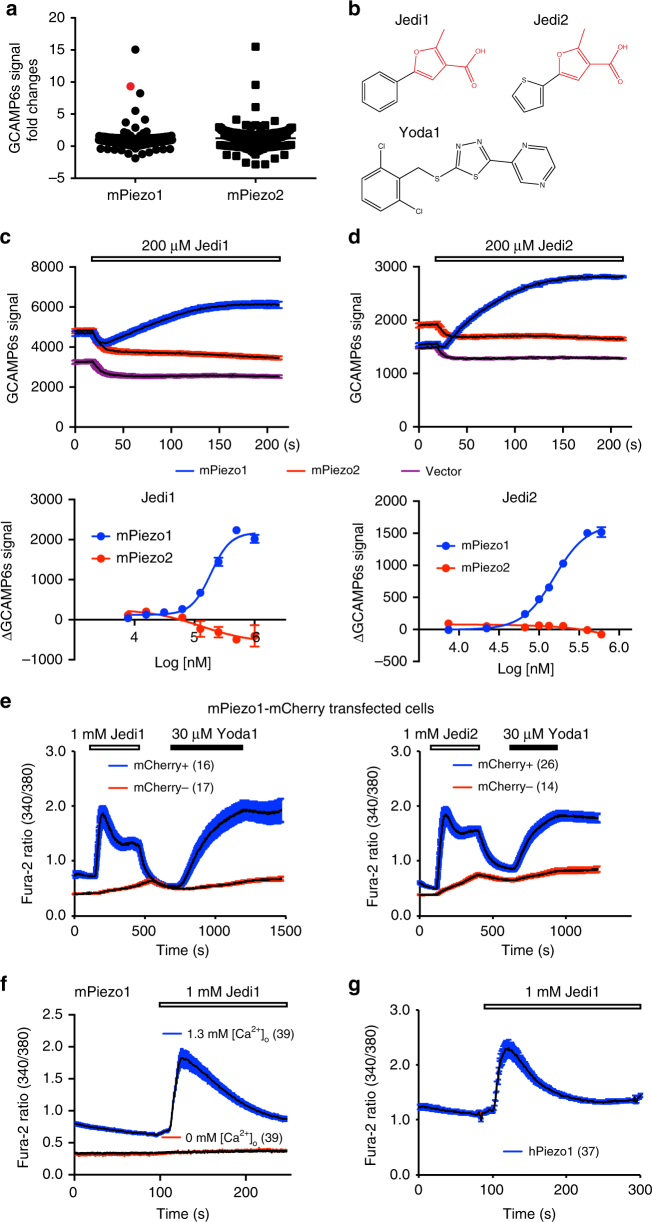
Identification of Jedi1 and Jedi2 that evoke Piezo1-mediated Ca^2+^ influx. **a** A summary view of fold changes of the GCAMP6s fluorescent signal of HEK293T cells co-transfected with either mPiezo1 or mPiezo2 and GCAMP6s in response to 96 compounds assayed by FLIPR in a 96-well format. The red dot represents a compound that evoked specific response in mPiezo1- but not in mPiezo2-transfected cells. **b** The chemical structures of Jedi1, Jedi2, and Yoda1. **c**, **d** Top panels: representative FLIPR traces showing the GCAMP6s fluorescent signal changes of HEK293T cells transfected with the indicated constructs in response to either 200 μM Jedi1 (left panel) or Jedi2 (right panel). The black trace represents the average response of 4-well repeats. Error bars are shown in colors. The addition of compound solution into the well caused an artifact drop of the fluorescent signal. The slow activation kinetics might be due to the slow Ca^2+^-binding kinetics of GCAMP6s. Lower panels: Jedi1 and Jedi2 dose response curves of mPiezo1- or mPiezo2-transfected cells (with GCAMP6s) as assayed by FLIPR. The compound-induced GCAMP6s signal change at each dose point from vector-transfected cells was correspondingly subtracted. The curve was fitted with a Boltzmann equation. Each data point represents mean ± s.e.m., *n* = 4 wells. **e** Representative Fura-2 ratio (340/380) traces obtained from single-cell Ca^2+^ imaging of mPiezo1-mCherry-transfected HEK293T cells in response to the indicated conditions. The black traces show the average responses of the mCherry-positive or -negative cells imaged from the same coverslip. The number of cells used for averaging is indicated. Error bars are shown in colors. **f**, **g** Single-cell Fura-2 Ca^2+^ imaging experiments showing the average response of mPiezo1 (**f**)- or human Piezo1 (hPiezo1) (**g**)-expressing cells in response to the indicated conditions. The number of cells used for averaging is indicated. Similar results were obtained from at least three experiments for data **e**–**g**

Pharmacological compounds are invaluable tools for probing the structure-function relationship of ion channels. To identify specific chemical activators of Piezo channels, we have used the fluorescent imaging plate reader (FLIPR) in a 96-well format to screen about 3000 compounds for those that can evoke Ca^2+^ increase in human embryonic kidney 293T (HEK293T) cells co-transfected with either mPiezo1 or mPiezo2, and the genetically encoded Ca^2+^ reporter GCAMP6s (high Ca^2+^ sensitivity but slow kinetics)^43^. We initially selected compounds that evoked Ca^2+^ response in either Piezo1- or Piezo2-transfected cells and subsequently ruled out those nonspecific hits that caused Ca^2+^ response in Piezo1-, Piezo2-, and vector-transfected cells (Fig. 2a). Eventually, we identified Jedi1 and Jedi2 as Piezo1 chemical activators, but no specific Piezo2 activators.

Jedi1 and Jedi2 are small molecules with molecular weights of 202.81 and 208.23 Da, respectively. They share a common structural motif of 3-carboxylic acid methylfuran (the part highlighted in red in Fig. 2b), indicating that this motif might account for their activating effect on Piezo1. Jedi1/2 have no structural similarity to the previously identified Piezo1 activator Yoda1^42^ (Fig. 2b), implying distinct activation mechanisms. The existence of the 3-carboxylic acid methylfuran motif in Jedi1/2 may explain their relatively better water solubility (up to ~2 mM) than Yoda1 (up to ~30 μM)^42^.

Both Jedi1 and Jedi2 specifically elicited dose-dependent responses in mPiezo1-transfected cells, but not in mPiezo2- or vector-transfected cells (Fig. 2c, d). In line with Jedi possessing a common structural motif, Jedi1 and Jedi2 had a comparable EC_50_ of ~200 and 158 μM, and Hill coefficient of ~1.5 and 1.6 for mPiezo1, respectively (Fig. 2c, d). Compounds at doses of sub-mM to mM range are commonly used for studying ion channels involved in somatosensation such as the transient receptor potential (TRP) channels^44^. For instance, sub-mM of menthol and mM of camphor have been used to activate TRPM8 and TRPV3, respectively. We further characterized the Jedi-evoked Ca^2+^ responses using single-cell Ca^2+^ imaging of Fura-2, a ratiometric Ca^2+^ dye (Fig. 2e–g). Both Jedi1 and Jedi2 caused an increase in Ca^2+^ in mCherry-positive HEK293T cells transfected with the mPiezo1-mCherry construct, which also responded to Yoda1 (Fig. 2e). Furthermore, removing extracellular Ca^2+^ abolished the response, suggesting that Jedi1/2 caused Ca^2+^ influx, rather than Ca^2+^ release from the endoplasmic reticulum Ca^2+^ store (Fig. 2f). Human Piezo1 (hPiezo1) responded to Jedi1 as well (Fig. 2g). Notably, the Jedi-induced response showed a rapid activation, apparent decay, and fast reversibility (Fig. 2e–g). In contrast, the Yoda1-induced response had slow activation, no decay, and poor reversibility (Fig. 2e). These data imply that Jedi and Yoda1 have distinct activation mechanisms.

### Jedi elicits Piezo1-mediated currents

**Fig. 3:**
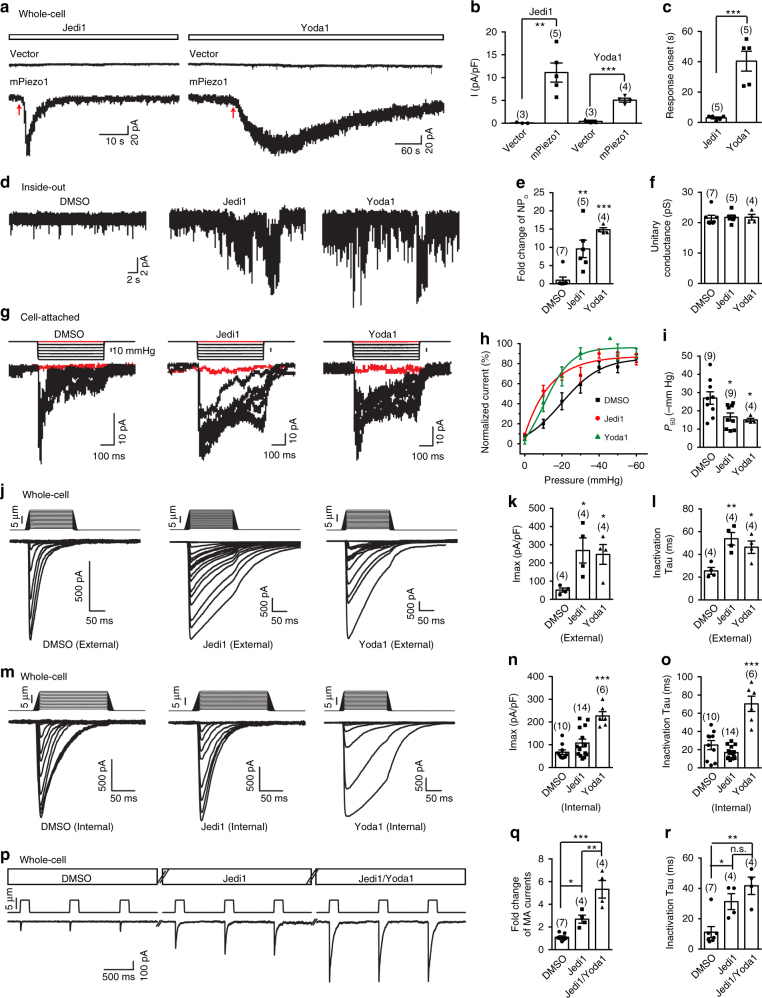
Electrophysiological effects of Jedi1 and Yoda1 on Piezo1. **a** Representative whole-cell current traces in response to either 1 mM Jedi1 or 30 μM Yoda1, which was puffed onto the recorded cells. The red arrows indicate the onset of the current. **b**, **c** Scatter plot of the current amplitudes (**b**) or onset (**c**) induced by the compound. Statistical significance was assessed using the unpaired Student’s *t*-test. **d** Representative channel activities of mPiezo1-expressing cells in the inside-out patch configuration at −80 mV. The compounds were in the pipette solution. **e**, **f** Scatter plot of the NP_o_ (**e**) or unitary conductance (**f**). Statistical significance was assessed using one-way ANOVA with Dunn’s comparison to DMSO. **g** Representative stretch-activated currents at −80 mV from mPiezo1-expressing cells. The red traces show the single-channel activities in the absence of externally applied force (Supplementary Fig. 1d). **h** Pressure-current relationships fitted with a Boltzmann equation. **i** Scatter plot of the *P*_50_ calculated from fit of the pressure-current relationship of individual recordings with a Boltzmann equation. Statistical significance was assessed using one-way ANOVA with Dunn’s comparison to DMSO. **j**, **m** Representative traces of poking-induced inward currents at −60 mV with the indicated compound present either in the extracellular (**j**) or internal (**m**) recording buffer. **k**, **n** Scatter plot of the maximal poking-induced currents. Statistical significance was assessed using one-way ANOVA with Dunn’s comparison to DMSO. **l**, **o** Scatter plot of the inactivation Tau of the poking-induced currents. Statistical significance was assessed using one-way ANOVA with Dunn’s comparison to DMSO. **p** Three consecutive poking-induced current traces that reached the steady state under DMSO, 200 μM Jedi1, and 200 μM Jedi1/30 μM Yoda1, respectively, are shown. **q**, **r** Scatter plot of the fold change (**q**) or inactivation tau (**r**) of the poking-induced currents. Statistical significance was assessed using one-way ANOVA with Tukey’s multiple comparisons test. Each bar represents mean ± s.e.m., and the recorded cell number is labeled above the bar. ***P < 0.001, **P < 0.01, *P < 0.05

We next used patch-clamp to characterize the electrophysiological effects of Jedi on Piezo1. In a whole-cell patch configuration, while in a gap-free recording mode, we found that puff-application of Jedi1 or Yoda1 to the mPiezo1-mRuby2-expressing HEK293T cells evoked inward currents (Fig. 3a, b). In contrast, neither compound triggered currents from vector-transfected cells (Fig. 3a, b). These data demonstrate agonistic effect of Jedi1 and Yoda1 on Piezo1, consistent with their ability to trigger Piezo1-dependent Ca^2+^ influx. Furthermore, in line with our single-cell calcium-imaging results (Fig. 2e), the Jedi1-evoked currents have a more rapid onset and faster decay than Yoda1-induced currents (Fig. 3a, c), further supporting that Jedi1 and Yoda1 have distinct activation mechanisms.

To test whether the activating effect of Jedi1 and Yoda1 on Piezo1 requires intracellular mediators, we performed inside-out patch recordings with the compounds present inside the pipette solution. Jedi1 and Yoda1 caused 9.6 ± 2.4 and 14.8 ± 0.6 fold change of the open probability (NP_o_), respectively (Fig. 3d, e). Similarly, Jedi2 also caused significant increase in the opening probability of Piezo1 (Supplementary Fig. 1a, b). Furthermore, the unitary conductance measured at −80 mV was not altered by any of the compounds (Fig. 3f and Supplementary Fig. 1c). Together, these data suggest that the agonistic effects of Jedi1/2 and Yoda1 on Piezo1 are independent of intracellular mediators.

### Jedi1 potentiates Piezo1 through the extracellular side

We next assessed the effect of Jedi1 and Yoda1 on stretching-induced Piezo1 currents using a cell-attached patch configuration coupled with a pressure clamp. In the absence of externally applied pressure (however, it should be noted that membrane tension might exist in cell-attached patches), patches with either Jedi1 or Yoda1 present inside the pipette solution exhibited more spontaneous single-channel openings than patches with the dimethylsulfoxide (DMSO) control (4.7 ± 1.4 and 3.1 ± 1.3 fold change of NP_o_ caused by Jedi1 and Yoda1, respectively; Supplementary Fig. 1d, e). This is consistent with the agonistic effect of Jedi1 and Yoda1 on Piezo1 (Fig. 3a–e). Neither Jedi1 nor Yoda1 altered the maximal stretching-induced Piezo1 current (Supplementary Fig. 1f), indicating no change in the number of channels (~40 channels) present in the membrane patch. Consistent with the previous report^42^, Yoda1 caused a significant leftward shift of the pressure-current relationship (Fig. 3g, h). Jedi1 caused a similar effect (Fig. 3g, h). The measured *P*_50_ (the pressure required for half maximal activation) is −27.0 ± 3.4, −16.7 ± 2.2, and −15.1 ± 0.9 mm Hg for DMSO, Jedi1, and Yoda1, respectively (Fig. 3i). Jedi1 and Yoda1 appear not to significantly affect the slope sensitivity (*s*) of mPiezo1 (14.1 ± 0.7, 9.8 ± 1.9, and 8.0 ± 2.4 mm Hg for DMSO-, Jedi1-, and Yoda1-treated cells, respectively), which is comparable to that of human Piezo1 (7–8 mm Hg)^45^. Collectively, these data suggest that Jedi1 potentiates the mechanosensitivity of Piezo1.

We next examined the effect of Jedi on Piezo1-mediated whole-cell currents evoked via poking the plasma membrane with a piezo-driven blunted glass pipette. Upon the application of either Jedi1 or Jedi2, the poking-induced whole-cell currents of mPiezo1-mRuby2-expressing cells were increased 3.8 ± 0.5 and 5.4 ± 0.7 folds (Supplementary Fig. 1g, h), suggesting potentiation of Piezo1-mediated mechanically activated currents. We further examined the step-wise poking-induced currents of Piezo1 in the presence of DMSO, Jedi1, and Yoda1. When applied to the extracellular side, Jedi1 significantly enhanced the poking-induced maximal currents (Imax) and slowed the inactivation tau to a similar extent as Yoda1 did for Piezo1 (Fig. 3j–l). In contrast, when applied to the intracellular side, Jedi1 had no effect (Fig. 3m–o). Intracellular application of Yoda1 remained effective in enhancing the poking currents and slowing the inactivation (Fig. 3m–o). These data suggest that Jedi1 potentiates Piezo1-mediated poking currents from the extracellular side, consistent with its hydrophilic property. Furthermore, co-application of Jedi1 and Yoda1 caused a synergistic effect in potentiating the Piezo1 poking currents (Fig. 3p–r). These data further demonstrate that Jedi1 and Yoda1 act through distinct mechanisms to modulate Piezo1.

### Jedi and Yoda1 bind to the fragment of residues 1–2190

**Fig. 4:**
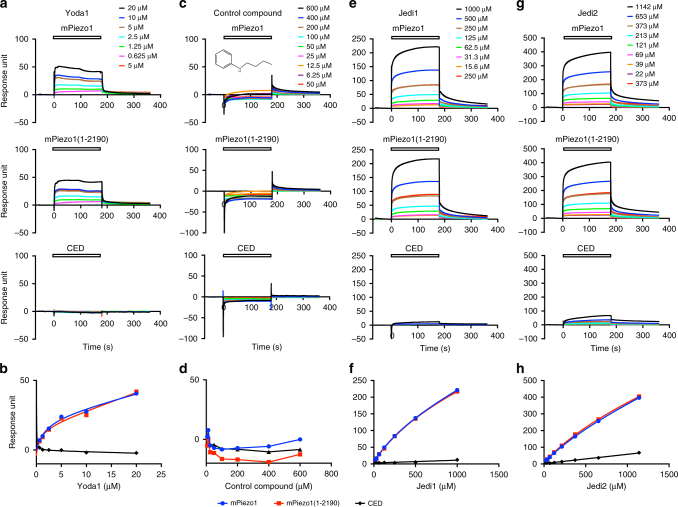
Jedi1/2 and Yoda1 bind to the fragment of residues 1–2190. **a**, **c**, **e**, **g** Representative SPR traces of the immobilized purified mPiezo1 (top panel), mPiezo1(1–2190) (middle panel), and CED (lower panel) in response to the series of concentrations of the indicated compounds measured with the affinity mode. To validate the reproducibility of the immobilized proteins regenerated after each dose point, a same dose (5, 50, 250, and 373 μM for Yoda1, control compound, Jedi1, and Jedi2, respectively) was re-tested. As exemplified in **e**, the red line overlaps with the brown line, indicating the reproducibility of the immobilized proteins for the assay. **b**, **d**, **f**, **h** The resulting dose-binding curve of the indicated compounds to the indicated proteins. The curves in **b**, **f**, and **h** were fitted with a total-binding equation. Similar results were obtained from two to eight independent experiments

We next employed the surface plasmon resonance (SPR) binding assay to examine whether Jedi1/2 bind directly to purified mPiezo1 proteins solubilized in the C_12_E_10_ detergent (Supplementary Fig. 2a, b). Based on an amine-coupling method, Piezo1 proteins (50 μg/ml) were optimally immobilized onto a research-grade CM5 sensor chip by using a buffer containing 10 mM sodium acetate (pH 5.0), 150 mM NaCl, 0.026% C_12_E_10_, and 0.05% Surfactant P20. Negative-staining electron microscopy revealed that the low pH buffer condition appeared not to cause notable changes in the integrity of the Piezo1 proteins (Supplementary Fig. 2c). Yoda1 has been shown to activate purified Piezo1 proteins reconstituted into lipid bilayers^42^, indicating a direct binding mechanism. In line with this, real-time SPR binding assay showed that Yoda1 bound to the immobilized mPiezo1 proteins with a *K*_d_ of 45.6 ± 14.3 μM (Fig. 4a, b), close to its estimated EC_50_ of ~17.1 μM^42^. These data not only provide direct evidence that Yoda1 binds to Piezo1 proteins but also validate the SPR binding assay for Piezo1-compound interaction. Importantly, we found that Jedi1 and Jedi2 bind to the mPiezo1 proteins with a *K*_d_ of 2754 ± 425 and 2770 ± 178 μM, respectively (Fig. 4e–g). In contrast, a control compound, which was present in the screened compound library but did not activate Piezo1, and has a benzene group but lacks the 3-carboxylic acid methylfuran motif possessed by Jedi1/2, showed no binding (Fig. 4c, d), indicating that the binding of Yoda1 and Jedi1/2 to Piezo1 is not due to nonspecific binding. The measured *K*_d_ of Jedi1 and Jedi2 is much higher than the EC_50_ assayed by the Ca^2+^ responses as shown in Fig. 2b. Given the hydrophilic nature of Jedi1/2, it is possible that the amine-based attachment of the purified Piezo1 protein to the SPR sensor chip might affect the binding. Nevertheless, together with the observation that Jedi1/2 act through the extracellular side of Piezo1 independently of intracellular mediators (Fig. 3), these binding results suggest that Jedi1/2 act directly on Piezo1. Nevertheless, given their low binding affinity, we could not totally exclude the possibility that Jedi1/2 might indirectly activate Piezo1 in the cell.

Since Jedi1/2 act through the extracellular side of Piezo1, we asked if it might bind to the C-terminal extracellular domain (CED; 2214–2457) that trimerizes to form the extracellular Cap-structure of the central ion-conducting pore module (Fig. 1)^8^. However, none of Jedi1/2, Yoda1, and the control compound bound to the purified CED proteins (without the use of the C_12_E_10_ detergent since they are soluble proteins; Fig. 4a–h). We next purified the large N-terminal fragment containing residues 1–2190 [mPiezo1(1–2190)] using the same purification procedure for mPiezo1. The fast protein liquid chromatography (FPLC) profile of the purified mPiezo1(1–2190) proteins revealed that they might exist in multiple oligomeric states, including trimers (Supplementary Fig. 2d, e). We used the FPLC fraction corresponding to the trimeric proteins (peak 2 of Supplementary Fig. 2d) for SPR binding assay. Jedi1/2 and Yoda1 bound to the mPiezo1(1–2190) proteins in a virtually identical fashion as to mPiezo1 (Fig. 4a, b, e–h). In contrast, the control compound did not show any binding (Fig. 4c, d). Collectively, these data suggest that Jedi1/2 and Yoda1 bind to the fragment of residues 1–2190, which forms the peripheral blade-beam structure and contains key mechanotransduction components^7,8,39^. Given that Jedi1/2 function through the extracellular side, we propose that Jedi1/2 bind to the extracellular loop regions of the blade. The distant location of the Jedi action region relative to the pore indicates a distinct long-distance allosteric gating of Piezo1.

### L15-16 and L19-20 are essential for Jedi activation

**Fig. 5:**
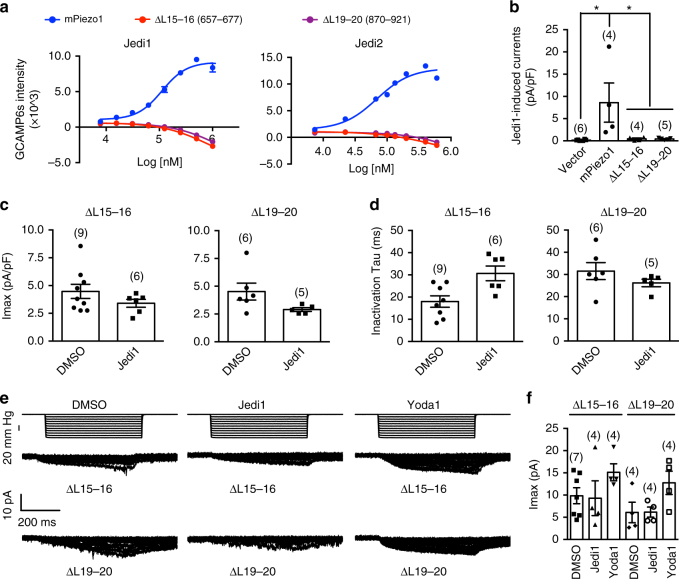
L15-16 and L19-20 are essential for Jedi activation of Piezo1. **a** Dose response curves of HEK293T cells transfected with GCAMP6s and the indicated constructs in response to the specified compounds as assayed by FLIPR. The curves were fitted with a Boltzmann equation. Each data point represents mean ± s.e.m., *n* = 4 wells. **b** Scatter plot of the chemical-induced currents from Piezo1-KO-HEK cells transfected with the indicated constructs. Statistical significance was assessed using one-way ANOVA with multiple comparison. **c** Scatter plot of the poking-induced Imax. Statistical significance was assessed using unpaired, two-tailed Student’s *t*-test. **d** Scatter plot of the inactivation tau of the poking-induced currents. Statistical significance was assessed using unpaired, two-tailed Student’s *t*-test. **e** Representative stretching-activated currents of cells recorded at −80 mV. **f** Scatter plot of the maximal stretching-activated currents. Statistical significance was assessed using one-way ANOVA with Dunn’s comparison to the DMSO-treated group. Each bar represents mean ± s.e.m., and the recorded cell number is labeled above the bar. *P < 0.05

We asked whether the extracellular loops of L15-16 and L19-20, which are critically involved in the mechanical activation of Piezo1^39^, is required for Jedi activation. Remarkably, Jedi1/2 evoked no dose-dependent Ca^2+^ responses from cells transfected with the loop deletion mutants (replaced with a poly-glycine GGGGG linker)^39^ ΔL15-16 (657-677), and ΔL19-20 (870–921; Fig. 5a). Jedi1 also failed to elicit either whole-cell currents (Fig. 5b) or potentiation of poking- (Fig. 5c, d) or stretching-induced currents from the mutant-transfected cells (Fig. 5e, f). These results demonstrate that the two loop regions are essential for Jedi-induced activation of Piezo1. In contrast, Yoda1 retained its ability to evoke Ca^2+^ responses and to potentiate the poking-induced currents from the mutant-transfected cells^39^, further indicating distinct activation mechanisms of Piezo1 by these two classes of activators. Intriguingly, Yoda1 failed to potentiate stretching-induced currents from the mutants (Fig. 5e, f), raising the possibility that Piezo1 might employ distinct molecular bases for responding to different forms of mechanical stimulation such as stretching and poking.

We asked whether these two regions might form the binding site for Jedi1/2. FPLC profiles of the purified ΔL15-16 and ΔL19-20 proteins revealed the peak fraction that corresponded to trimeric Piezo1 complexes, suggesting normal trimerization of the mutant proteins (Supplementary Fig. 3a). Further SPR binding assay found that ΔL15-16 and ΔL19-20 had similar binding to Jedi1/2 and Yoda1 as wild-type mPiezo1 did (Supplementary Fig. 3b-g). Thus, rather than being the major binding site of Jedi1/2, the two extracellular loops form key transduction sites for mediating Jedi-induced activation of Piezo1.

### L1342 and L1345 are required for chemical activation

**Fig. 6:**
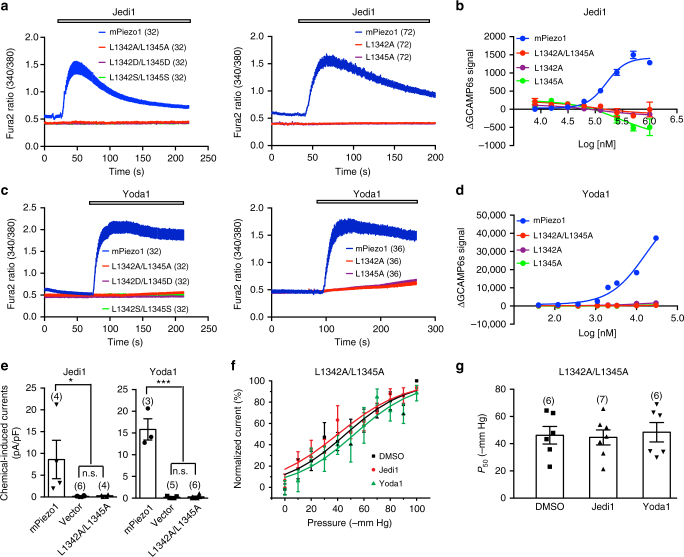
L1342 and L1345 are essential for Jedi1 and Yoda1 activation of mPiezo1. **a**, **c** Representative single-cell Fura-2 Ca^2+^ imaging traces showing the average responses of cells transfected with the indicated constructs in response to 1 mM Jedi1 (**a**) or 30 μM Yoda1 (**c**). **b**, **d** Dose response curves of cells transfected with the indicated constructs and GCAMP6s in response to Jedi1 (**b**) or Yoda1 (**d**). The curves were fitted with a Boltzmann equation. Each data point represents mean ± s.e.m., *n* = 4 wells. **e** Scatter plot of the chemical-induced currents from the Piezo1-KO-HEK cells transfected with the indicated constructs. Statistical significance was assessed using one-way ANOVA with multiple comparison test. **f** Pressure-current relationships of the stretch-induced currents of the L1342A/L1345A mutant under the indicated compound conditions. The curves were fitted with a Boltzmann equation. **g** Scatter plot of the *P*_50_ calculated from fit of the pressure-current relationship of individual recordings with a Boltzmann equation. Statistical significance was assessed using one-way ANOVA with multiple comparison test. Data shown as mean ± s.e.m., and the recorded cell number is labeled above the bar. *P < 0.05, ***P < 0.001

The L1342A/L1345A mutant has impaired mechanical activation, and therefore L1342 and L1345 have been proposed to serve as the pivot for allowing the beam to form a lever-like apparatus for mechanotransduction^39^. We asked whether these two residues might also be involved in Jedi-mediated activation of Piezo1. We mutated L1342 and L1345 to different amino acids, resulting in the following mPiezo1 mutants: L1342A/L1345A; L1342D/L1345D; L1342S/L1345S; L1342A; and L1345A. Strikingly, all the mutants completely lost both Jedi1- and Yoda1-evoked Ca^2+^ responses as assayed by either single-cell Ca^2+^ imaging or FLIPR (Fig. 6a–d). Furthermore, Jedi1 and Yoda1 neither elicited inward currents (Fig. 6e) nor affected the stretching-current relationship (Fig. 6f) and *P*_50_ of the L1342A/L1345A mutant (Fig. 6g). In contrast, mutating the Q1344 residue (Q1344A) did not affect Jedi1- or Yoda1-induced Ca^2+^ responses (Supplementary Fig. 4a, b), demonstrating the specific requirement of L1342 and L1345 in this region for Jedi1 and Yoda1 responses. The purified L1342A/L1345A mutant proteins retained the ability to bind to Jedi1/2 and Yoda1 as assayed by SPR (Supplementary Fig. 5), indicating that these two residues did not form the binding site for Jedi1/2 and Yoda1. Together, our data demonstrate that L1342 and L1345 play an essential role in Jedi- and Yoda1-induced activation of Piezo1.

## Discussion

The Piezo1 ion channel is the founding member of the mammalian MS cation channels that play crucial roles in various mechanotransduction processes^5,6^. Thus, it is important to thoroughly understand its mechanogating and ion permeation mechanisms. Toward this, a major step is to dissect out the key functional components and mechanotransduction pathways within the gigantic Piezo1 channel complex (~0.9 megadalton), which forms a unique three-bladed, propeller-shaped structure, comprising a central ion-conducting pore and three surrounding blade-beam structures^7,8,39^. Here, via identifying and characterizing a novel set of Piezo1 chemical activators, we have revealed that the blade-beam-structure constitutes a designated transduction pathway for propagating chemical-induced action from the extracellular side of the distal blade to the central pore (Fig. 1). These findings support the working model that Piezo1 might employ its peripheral blade-beams as lever-like apparatuses for effective long-range allosteric gating of Piezo1^39^.

Our electrophysiological characterizations and SPR measurements suggest that Jedi1/2 might act through the extracellular regions of the peripheral blade, which is formed by the large region containing residues 1–2190 (Figs. 3 and 4). Remarkably, the mPiezo1-ΔL15-16 (657–677) and mPiezo1-ΔL19-20 (870–921) deletion mutants completely lost their responsiveness to Jedi1/2 (Fig. 5). However, these two extracellular loops do not form the major binding sites for Jedi1/2 (Supplementary Fig. 3). Together with their critical role in mechanical activation of Piezo1^39^, they thus serve as key transduction sites for mechanical and chemical activation of Piezo1. Given that Jedi1/2 initiate the activation process from the extracellular side of Piezo1, it is likely that the conformational change upon their binding is sequentially transduced along their binding sites, the extracellular L15-16/L19-20 and then the intracellular beam. Characteristically, nine tandem THU repeats are organized in serials to form the highly curved blade structure^39^. L15-16 and L19-20 are respectively located in the THU4 and THU5 (Fig. 1). Thus, we hypothesize that the extracellular loop regions of the upstream THU1-THU3 (L3-4, L7-8, and L11-12) might contain Jedi-binding sites. In line with this hypothesis, previous studies have identified MS domains located in L3-4 (THU1) and L7-8 (THU2)^46^. Unfortunately, deleting these extracellular loops (replaced with a GGGGG linker) affects the expression and proper localization of the mutant proteins in the plasma membrane, preventing us from characterizing their responsiveness to Jedi1/2^39^.

The 90 Å-long intracellular beam physically connects the distal blade to the pore, thus might serve as an ideal structure for mechanical transmission from the distal THUs to the central pore. Indeed, like the blade, the beam displays uneven movement with large motion at the distal end while subtle movement at the proximal end^39^. As a whole, the motion feature of the blade-beam is reminiscent of a lever apparatus. In line with its functional importance, mutating L1342 and L1345, which are located in the proximal end of the beam, not only completely abolished Jedi1 activation of Piezo1 (Fig. 6) but also affected the mechanical activation of Piezo1^39^. This is striking given that Jedi1 acts at the extracellular side. These results provide strong evidence in supporting the model that the beam functions as a key transduction component downstream of the extracellular transduction components of L15-16/L19-20.

L1342/L1345 might serve as a pivot for allowing the beam to form an effective lever for coupling the distal bade and the central pore of Piezo1^39^ (Fig. 1). According to the lever principle, the closer localization of the pivot to the central pore can lead to an effective amplification of the input force acting on the distal blades to provide a greater force harnessed to gate the central ion-conducting pore. At the same time, it enables the conversion of a large conformational change of the distal blades to a relatively slight opening of the central pore, ensuring cation-selective permeation. Furthermore, the lever-type mechanism would be suited for responding to large-scale changes in membrane curvature. This might be particularly important for Piezo1 as its distal THU4-6 adopt a highly curved configuration^39^ (Fig. 1). Thus, a lever-like mechanotransduction mechanism may enable Piezo1 to function as an effective mechanotransducer for converting mechanical stimuli into cationic currents. Remarkably, three sets of such blade-beam-constituted lever-like apparatuses are further assembled into the gigantic three-bladed, propeller-like machinery of Piezo1, which might enable it to function as one of the most sophisticated mechanotransducers. Indeed, this mechanogating mechanism appears to fit very well with the observation that Piezo channels are effectively gated by moving extracellular matrix in the scale of ~10 nm at the cell-substrate interface through an elegant pillar assay developed by Lewin and colleagues^34,35^.

Jedi and Yoda1 appear to activate and modulate Piezo1 via acting on different loci along the blade-beam gating pathway. The ΔL15-16 and ΔL19-20 mutants completely lost their responsiveness to Jedi1/2 (Fig. 5), but retained Yoda1-induced activation and potentiation of the poking currents^39^. In contrast, the L1342/L1345 mutants are insensitive to both Jedi and Yoda1 (Fig. 6). These data suggest that Jedi1/2 act on the upstream blade, while Yoda1 acts at the downstream beam. Besides pharmacological manipulation, we speculate that modulations of the blade-beam gating pathway by endogenous cellular components might represent an important mechanism for regulating Piezo1 mechanotransduction. In line with this, multiple phosphorylation sites have been identified in the beam-containing intracellular loop.

Piezo1 responds to different forms of mechanical stimulation such as poking and stretching. Our data suggest that Piezo1 might employ discrete mechanosensing and transducing components for poking and stretching. For instance, the ΔL15-16 and ΔL19-20 mutants appeared to completely lose their ability to generate stretching-induced currents, but remained able to produce residual poking currents^39^. Furthermore, Yoda1 preferentially potentiated their poking currents^39^, but not the stretching currents (Fig. 5). In contrast, the L1342A/L1345A mutant could still respond to stretching, but had reduced stretching sensitivity^39^, indicating the existence of other mechanotransduction pathways responsible for the remaining stretching sensitivity. Together, our data suggest that the extracellular loops of L15-16 and L19-20 might serve as upstream loci essential for stretching sensing and transduction, while the beam might function as one of multiple downstream transduction pathways. Unraveling the remaining mechanotransduction pathways in the future will clearly provide further insights into the mechanogating mechanisms of the Piezo channels.

## Methods

### FLIPR and compound screening

HEK293T cells were grown in Dulbecco’s modified Eagle medium containing 4.5 mg/ml glucose, 10% fetal bovine serum, and 1% penicillin/streptomycin. Cells were seeded in 50 μg/ml poly-d-lysine-coated 96-well plates (3 × 10^4^ cells/well) and allowed to grow for about 18 h, and then co-transfected with a total of 250 ng cDNA containing Piezo1 or Piezo2 and GCaMP6s using Lipofectamine 2000 (Invitrogen, Life Technology). Two days after transfection the cells were washed with buffer containing 1× Hanks’ balanced salt solution (HBSS; 1.3 mM Ca^2+^) and 10 mM HEPES (pH 7.2). The 96-well plates were then transferred to the FLIPR Tetra (Molecular Device) for monitoring the GCAMP6s fluorescence before and after addition of the compounds that were prepared in separate compound plates. For the initial screening, we screened about 3000 compounds derived from either the chemical library collected in Dr. Yu Rao’s lab, which is composed of different classes of heterocycles, including various drug scaffold or the Maybridge Ro3 Diversity Fragment Library purchased from Maybridge (Cambridge, UK), which consists of 2500 fragment compounds with a molecular weight below 350 and high rule of three compliance. Each compound was tested at a final concentration of 200 μM. Approximately 20 positive hits were selected for further testing in Piezo1-, Piezo2-, or vector-transfected cells. For subsequent characterizations, Jedi1 was purchased from the company of Alfa Aesar, while Jedi2 and Yoda1 were synthesized in the laboratories of Drs. Wei He and Liansuo Zu, respectively. See the Supplementary Experiment Procedures for the chemical synthesis. The dose response measurement by FLIPR was similar to previously described^39^.

### Fura-2 single-cell Ca^2+^ imaging

Fura-2 single-cell Ca^2+^ imaging was performed according to the previous protocol^47^. In brief, mCherry-Piezo1 cDNA (1 μg)-transfected HEK293T cells were plated onto 12-mm round glass coverslips, which were coated with poly-d-lysine and placed in 24-well plates, and subject to Fura-2 single-cell Ca^2+^ imaging about 36 h post transfection. Cells were washed with buffer containing 1× HBSS (1.3 mM Ca^2+^) and 10 mM HEPES (pH 7.2), incubated with 2.5 μM Fura-2 and 0.05% Pluronic F-127 (Life Technologies) for 30 min, then washed again with the previously mentioned buffer. The coverslip was mounted into an inverted Nikon-Tie microscope equipped with a CoolSNAP charge-coupled devise (CCD) camera and Lambda XL light box (Sutter Instrument), and mCherry-positive and -negative cells were selected for measurement of the 340/380 ratio with a ×20 objective (numerical aperture 0.75) using the MetaFluor Fluorescence Ratio Imaging software (Molecular Device).

### Whole-cell electrophysiology and mechanical stimulation

Protocols for HEK293T cell culture, transient transfection, and patch-clamp experiments with an Axopatch 200B amplifier (Axon Instruments) or HEKA EPC10 were essentially similar to how previously described^6,8^. For whole-cell patch-clamp recordings, recording electrodes had a resistance of 2–3 MΩ when filled with internal solution composed of (in mM) 133 CsCl, 1 CaCl_2_, 1 MgCl_2_, 5 EGTA, 10 HEPES (pH 7.3 with CsOH), 4 MgATP, and 0.4 Na_2_GTP. The extracellular solution was composed of (in mM) 133 NaCl, 3 KCl, 2.5 CaCl_2_, 1 MgCl_2_, 10 HEPES (pH 7.3 with NaOH), and 10 glucose. All experiments were carried out at room temperature. Currents were sampled at 20 kHz, filtered at 2 kHz using the Clampex 10.4 software (Axon Instruments) or Patchmaster software. Leak currents before mechanical stimulations were subtracted offline from the current traces.

Mechanical stimulation was delivered to the cell during the patch-clamp recording at an angle of 80° using a fire-polished glass pipette (tip diameter 3–4 μm) as previously described^5,6^. Downward movement of the probe toward the cell was driven by a Clampex-controlled piezo-electric crystal micro-stage (E625 LVPZT Controller/Amplifier; Physik Instrument). The probe had a velocity of 1 μm/ms during the downward and upward motion and the stimulus was maintained for 150 ms. A series of mechanical steps in 1 μm increments was applied every 20 s and currents were recorded at a holding potential of −70 mV. The poking distance shown in Fig. 3 includes the initial distance between the poking pipette and the cell, which cannot be accurately controlled, and the applied poking distance controlled by the Clampex-controlled piezo-electric crystal micro-stage. Thus, the actual poking distance on the cell membrane should be equal to the applied poking distance minus the initial distance between the poking pipette and the cell under recording. In the step-wise poking experiment, the patch was readily lost when the cell was broken at the end of the poking.

Stock solutions of Jedi1 (100 mM), Jedi2 (100 mM), and Yoda1 (30 mM) were solubilized in DMSO and diluted to the desired final concentrations as indicated in the figure legends using external or internal solutions. In Figs. 3a,  5b, and 6e, the compound solutions were puffed onto the cells using a multichannel perfusion system (MPS-2, World Precision Instruments) while continuously recording using a gap-free mode. In other conditions, the compounds were directly added to the recording chamber or pipette solution, and the currents were recorded within 10 min. In Figs. 5b and 6e, we used the Piezo1-KO-HEK cell line in which the endogenous *Piezo1* gene is disrupted.

### Cell-attached electrophysiology

Stretch-activated currents were recorded in the cell-attached patch-clamp configuration as previously described^8^. Currents were sampled at 20 kHz and filtered at 2 kHz. Pipette was filled with a solution consisting of (in mM) 130 NaCl, 5 KCl, 10 HEPES, 1 CaCl_2_, 1 MgCl_2_, and 10 TEA-Cl (pH 7.3, balanced with NaOH). The external solution used to zero the membrane potential consisted of (in mM) 140 KCl, 10 HEPES, 1 MgCl_2_, and 10 glucose (pH 7.3 with KOH). All experiments were done at room temperature. Membrane patches were stimulated with negative pressure pulses for 500 ms through the recording electrode using a Patchmaster-controlled pressure clamp HSPC-1 device (ALA-scientific). Stretch-activated channels were recorded at a holding potential of −80 mV with pressure steps from 0 to −100 mm Hg (−10 mm Hg increments), and 4–11 recording traces were averaged per cell for analysis. Current-pressure relationships were fitted with a Boltzmann equation of the form: *I*(*P*) = [1 + exp (−(*P *– *P*_50_)/*s*)]^−^^1^, where *I* is the peak of stretch-activated current at a given pressure, *P* is the applied patch pressure (in mm Hg), *P*_50_ is the pressure value that evoked a current value which is 50% of Imax, and *s* reflects the current sensitivity to pressure.

For inside-out patch-clamp recordings using an Axopatch 200B amplifier (Axon Instruments), the “extracellular” pipette solution consists of (in mM) the following: 130 NaCl, 5 KCl, 10 HEPES, 1 CaCl_2_, 1 MgCl_2_, and 10 TEA-Cl (pH 7.3 with NaOH), and the intracellular “bath” solution contains (in mM) the following: 140 KCl, 10 HEPES, 1 MgCl_2_, and 10 glucose (pH 7.3 with KOH). All experiments were done at room temperature. Currents were sampled at 20 kHz using Clampex 10.4 software (Axon Instruments) and filtered at 2 kHz. Additional offline filtering of 1 kHz was applied to the recordings for display. Single-channel activity in the presence or absence of Jedi or Yoda1 was continuously recorded for 5 min at −80 mV. The single-channel open probability (NP_o_) and unitary conductance of mPiezo1 were analyzed using the pClamp software built-in features. Current amplitudes of individual cells recorded at −80 mV were calculated from Gaussian fits to the current amplitude histograms.

### Molecular cloning

All the constructs were subcloned by using the one-step cloning kit following the instruction manual (Vazyme Biotech) as previously described^8,39^, and sequenced for validation of the desired mutations.

### Protein purification

The protein purification procedure was essentially similar to our previously described protocols^7^. Briefly, HEK293T cells were cultured in 150 mm × 25 mm dishes (a total of 50 dishes for each prep of protein purification) and transiently transfected with polyethylenimines (Polysciences), when the cells reached 80–90% of confluence. After 48 h, the transfected cells were collected, washed twice with phosphate-buffered saline (PBS), and homogenized in buffer A, containing 25 mM Na-PIPES, pH 7.2, 140 mM NaCl, 2 mM dithiothreitol (DTT), detergents CHAPS (1%) and C_12_E_9_ (0.1%), 0.5% (w/v) l-α-phosphatidylcholine (Avanti), and a cocktail of protease inhibitors (Roche) at 4 °C for 1 h. After centrifugation at 100 000 × *g* for 40 min, the supernatant was collected and incubated with glutathione–sepharose beads (GE Healthcare) at 4 °C for 3 h. The resin was washed extensively with buffer B, containing 25 mM Na-PIPES, pH 7.2, 140 mM NaCl, 2 mM DTT, 0.1% (w/v) C_12_E_9_ and 0.01% (w/v) l-α-phosphatidylcholine. The glutathione *S*-transferase-tagged Piezo1 wild-type or mutant proteins were cleaved off by PreScission Protease (Amersham-GE) in buffer B at 4 °C overnight, and applied to size-exclusion chromatography (Superpose-6 10/300 GL, GE Healthcare) in buffer C, containing 25 mM Na-PIPES, pH 7.2, 140 mM NaCl, 2 mM DTT plus 0.026% (w/v) C_12_E_10_. mPiezo1(WT), mPiezo1-Δ15-16, and mPiezo1-Δ19-20 displayed two well-resolved peaks. As previously characterized, the first peak of mPiezo1(WT) contains aggregated Piezo1 protein complexes, while the second one contains homogenous trimeric mPiezo1(WT) proteins. Comparison of the FPLC profiles between mPiezo1(WT) and Δ15-16 or Δ19-20 suggests that the second peak of the mutant proteins contains trimeric proteins. Thus, the second peak fraction of mPiezo1(WT), Δ15-16 or Δ19-20 was subjected to SPR binding experiments (Supplementary Fig. 3). For mPiezo1(1–2190), we detected two additional mPiezo1(1–2190)-containing peak fractions, which might correspond to trimeric and higher oligomeric complexes of the truncated mPiezo1, in addition to the first expected peak of aggregated proteins.

### SPR analysis

Real-time binding and analysis by SPR were conducted on BIAcore T200 instrument (GE Healthcare) at 25 °C. Proteins were immobilized on a research-grade CM5 sensor chip by amine-coupling methods. During the pH scouting process, 10 mM sodium acetate at various pH including 4.0, 4.5, 5.0, and 5.5 and protein concentrations varying between 10 and 100 μg/ml were screened before immobilization. Except pH 4.5 for mPiezo1-(1–2190), pH 5.0 was used for all other proteins, including mPiezo1, CED, Δ15-16, and Δ19-20. All proteins were diluted to 50 μg/ml in buffer containing 10 mM sodium acetate at the abovementioned pH, 0.026% C_12_E_10_ detergent, and 150 mM NaCl, except that the soluble CED proteins, which were diluted in 150 mM NaCl without detergent. During the immobilization process, the CM5 sensor was activated with NHS first, allowing the amine-containing proteins to be coupled to the sensor through NHS. Each CM5 sensor had 4 flow cells, and the flow cells 1 and 3 were often left blank as a reference. Diluted Piezo1 wild-type or mutant proteins (50 μg/ml) in their respective buffers were immobilized on the flow cell 2 or flow cell 4 to the similar level as indicated by the “response bound” parameter.

For data collection, compounds at various concentrations were diluted in a running buffer containing 1× PBS, 0.026% detergent C_12_E_10_, and 0.05% Surfactant P20 (GE Healthcare Bio-Sciences AB) and injected over the flow cells with at a 30 μl/min flow rate. The proteins and the compounds were allowed to associate for 180 s and then dissociate for 180 s. Data were analyzed with the BIAcore T200 evaluation software by fitting to a 1:1 affinity-binding model.

### Negative-staining electron microscopy

An aliquot of 4 μl Piezo1 (0.05 mg/ml) was applied to glow-discharged carbon-coated copper grids (200 mesh, Zhongjingkeyi, Beijing). After the grids were incubated at room temperature for 1 min, excessive liquid was absorbed by filter paper. Grids containing the specimen were stained by applying droplets of 2% uranyl acetate for 30 s and then air-dried. Micrographs were taken on a T12 microscope (FEI) operated at 120 kV, using a 4k × 4k CCD camera (UltraScan 4000, Gatan). Images of Piezo1 at pH 7.4 and pH 5.0 were taken at a nominal magnification of 49 000 times.

### Data availability

The data that support the findings of this study are available from the corresponding author upon request.

## Electronic supplementary material

Supplementary Information (PDF 5829 kb)(PDF 5421 kb)
