# Supplementary material for: A lever-like transduction pathway for long-distance chemical- and mechano-gating of the mechanosensitive Piezo1 channel
Source: Nat Commun. 2018 Apr 3;9:1300. doi: 10.1038/s41467-018-03570-9 (PMC5880808; doi:10.1038/s41467-018-03570-9)

“A lever-like transduction pathway for long-distance chemical- and mechano-gating of the mechanosensitive Piezo1 channel” by Wang et al.

Supplementary Figures

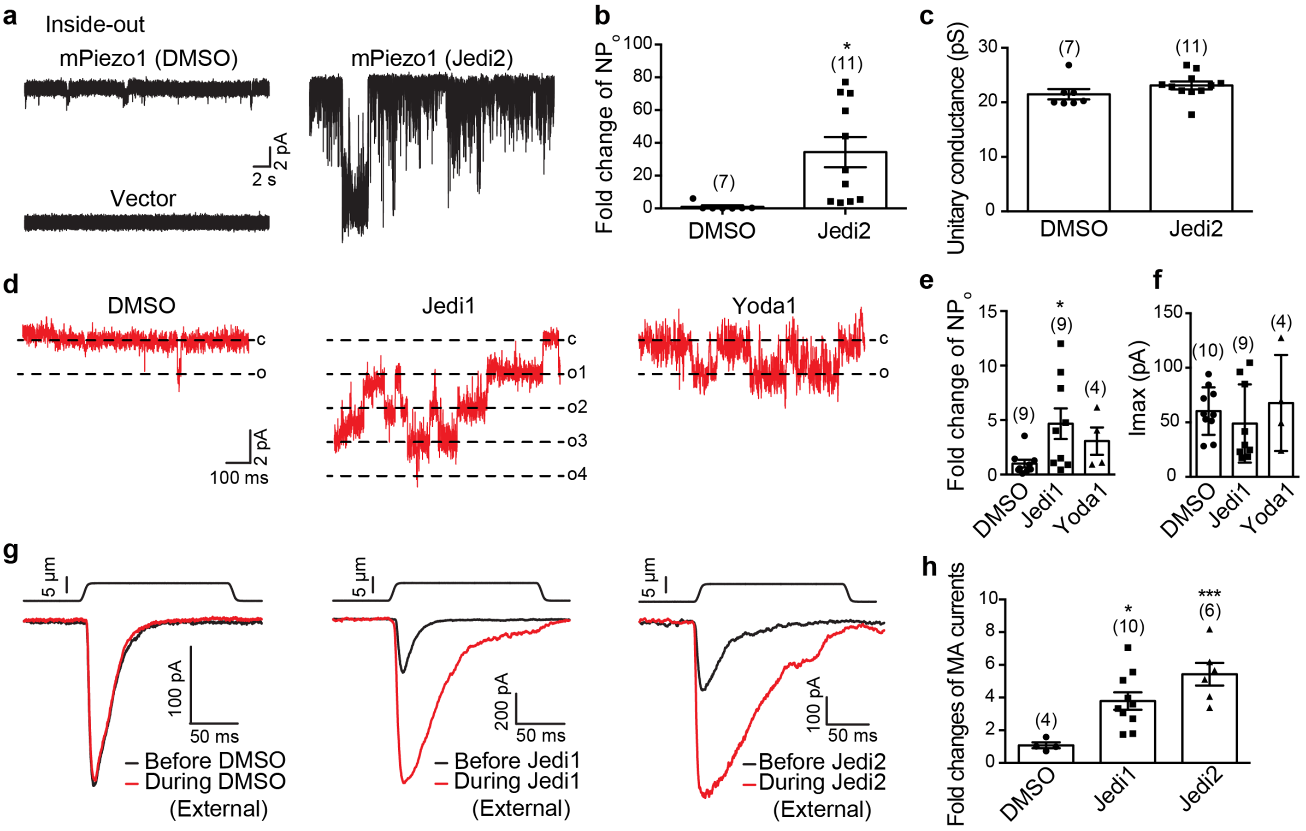

Supplementary Figure 1 Electrophysiological effects of Jedi1/2 and Yoda1 on Piezo1

a, Representative channel activities of cells transfected with mPiezo1 or the control vector in response to the indicated compounds in the absence of externally applied force under inside-out patch configuration at -80 mV. The indicated compounds were in the pipette solution.

b and **c**, Scatter plot of the fold-change of the single-channel open probability (NP_o) (**b**) or unitary conductance (**c**) of inside-out mPiezo1 channel activities. Statistical significance was assessed using unpaired Student's t-test.

d, Single-channel activities of mPiezo1-expressing cells in response to the indicated compounds in the absence of externally applied force recorded under cell-attached configuration (related to Fig. 3g).

e, Scatter plot of fold-change of NP_o of mPiezo1 in the absence of externally applied force under the indicated compound condition. Statistical significance was assessed using One-way ANOVA with Dunn's comparison to DMSO.

f, Scatter plot of the maximal stretch-induced currents of mPiezo1. Statistical significance was assessed using One-way ANOVA with Dunn's comparison to DMSO.

g, Representative poking-induced whole-cell currents recorded at -80 mV of mPiezo1-expressing cells before and during puffing application of 1 mM Jedi.

h, Scatter plot of the fold-change of the poking-evoked currents of mPiezo1. Statistical significance was assessed using One-way ANOVA with Dunn's comparison to DMSO.

Each bar represents mean ± s.e.m., and the recorded cell number is labeled above the bar. $^{*}P<0.05$, $^{***}P<0.001$.

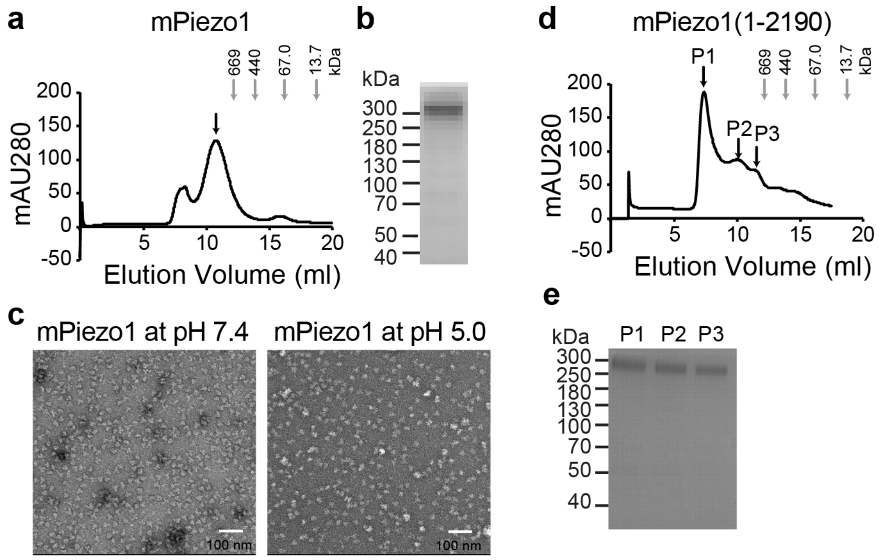

Supplementary Figure 2 Biochemical characterizations of the purified mPiezo1 and mPiezo1(1-2190) proteins.

a and **d**, FPLC profiles of mPiezo1 (**a**) and the mPiezo1(1-2190) mutant (**d**) proteins. The grey arrows indicate the molecular markers. The black arrows respectively indicate the peak fraction containing the mPiezo1 and mPiezo1(1-2190) mutant proteins.

b and **e**, Coomassie blue staining of the arrow indicated sample fractions shown in Panel **a** and **d** separated on 8% SDS-PAGE gels.

c, Negative staining of Piezo1 at pH 7.4 and pH 5.0 with a nominal magnification of 49,000 times.

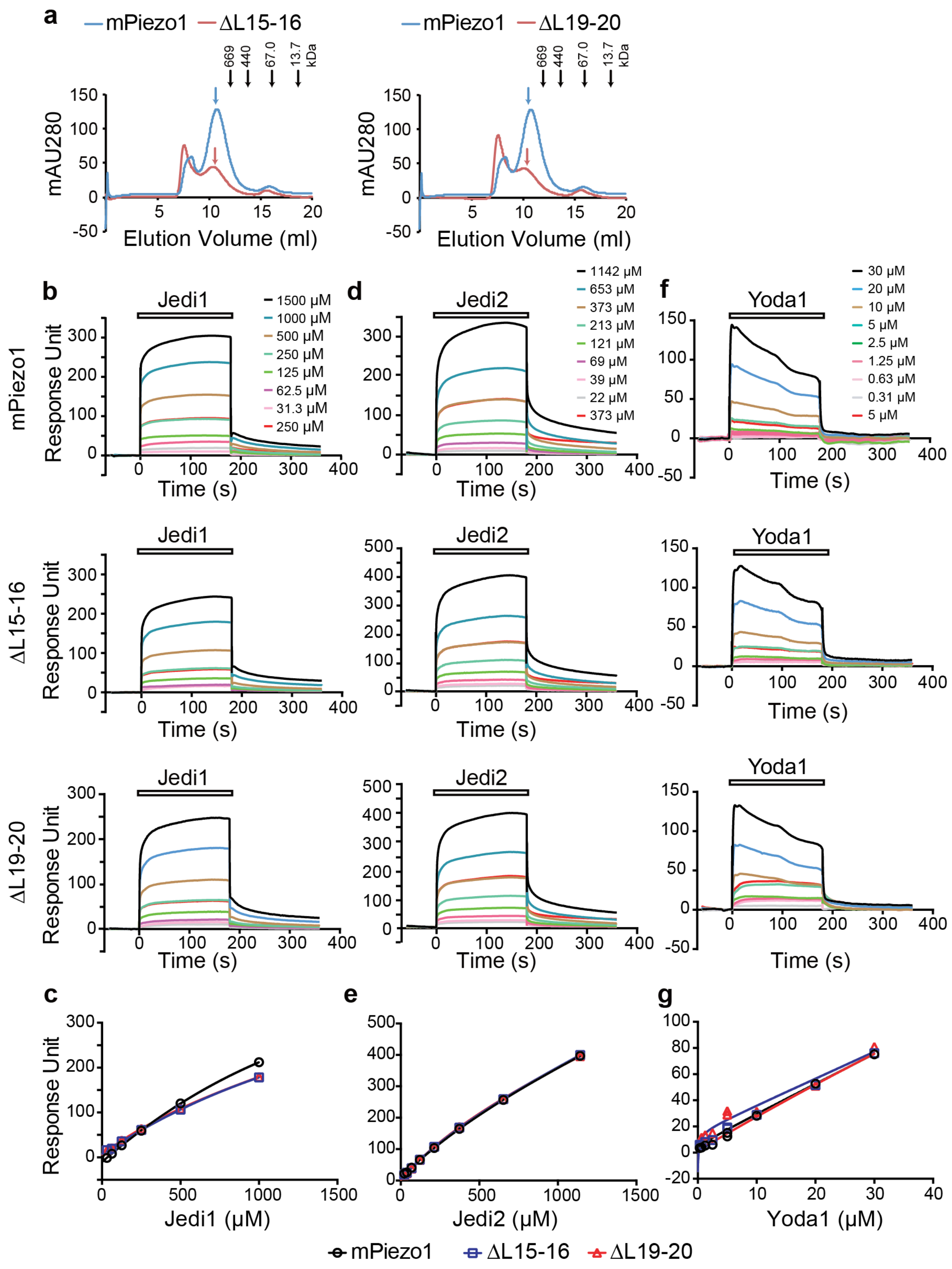

Supplementary Figure 3 Gel filtration and SPR binding.

a, FPLC profiles of mPiezo1 and the two deletion mutant proteins. The black arrows indicate the molecular markers. The blue and red arrows respectively indicate the peak fraction containing trimeric mPiezo1 and the mutant proteins.

b, **d**, **f,** Representative SPR traces of the indicated immobilized proteins in response to the series of concentrations of Jedi1, Jedi2 and Yoda1 measured with an affinity mode.

c, **e**, **g**, Dose response curves of mPiezo1 and the two mutants proteins to the indicated compounds.

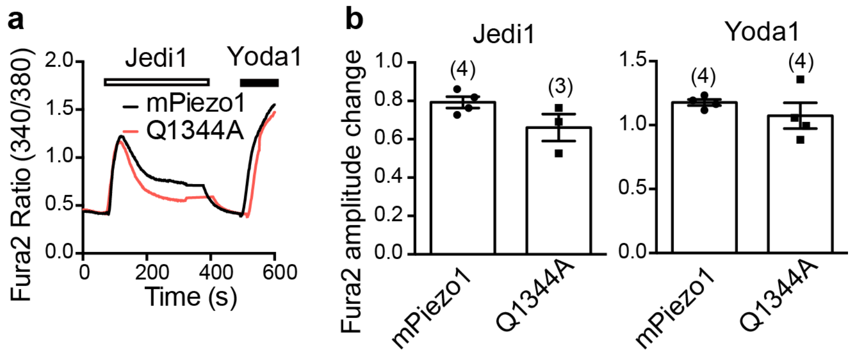

Supplementary Figure 4 Jedi1 and Yoda1 responses of the Q1344A mutant.

a, Fura-2 single cell Ca^{2+} imaging traces of cells transfected with the indicated constructs in response to 1 mM Jedi1 and 30 μM Yoda1.

b, Scatter plot of Jedi1- or Yoda1-induced Fura2 amplitude changes from mPiezo1- or Q1344A-expressing cells. Each bar represents mean ± s.e.m., and the n labeled above the bar represents number of coverslips. Statistical significance was assessed using unpaired Student's t-test.

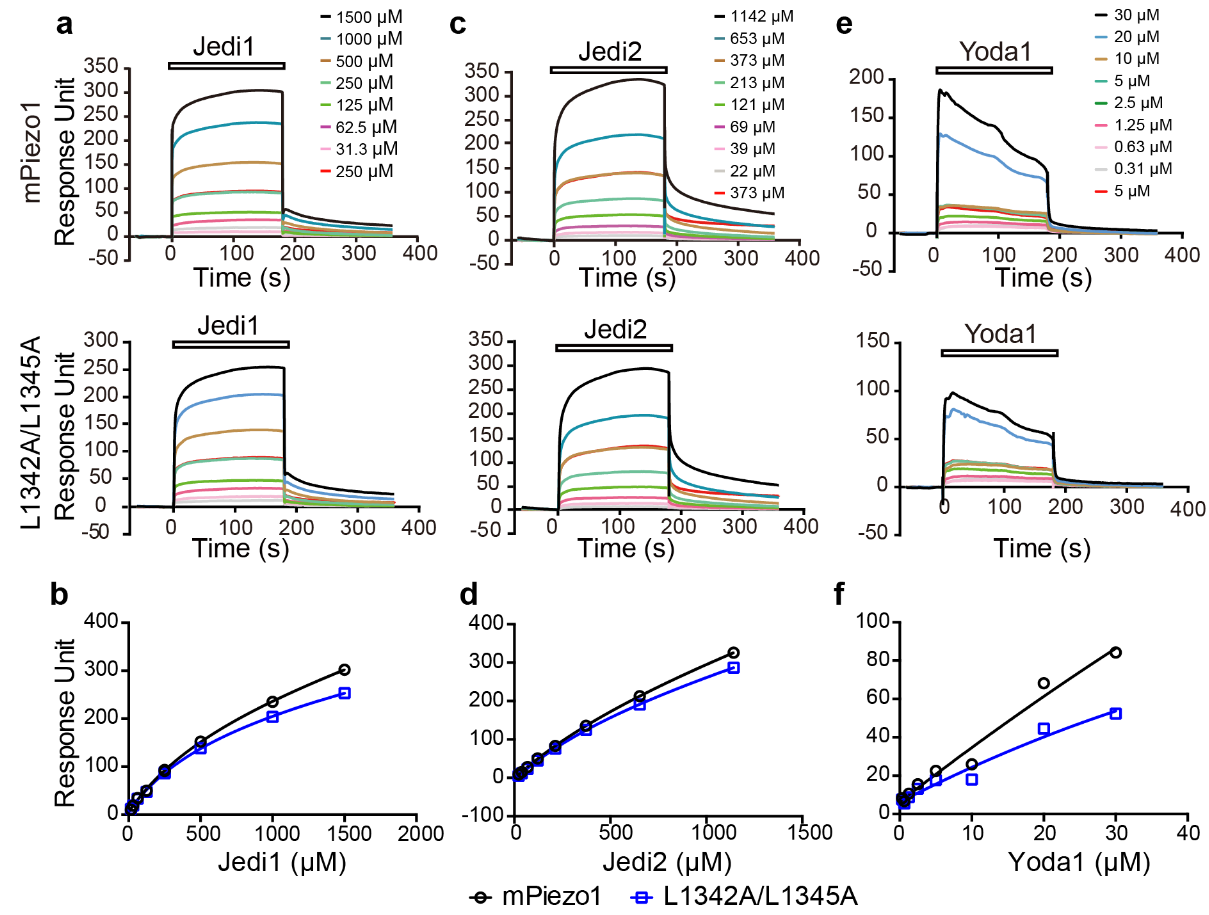

Supplementary Figure 5 SPR binding of the purified L1342A/L1245A mutant protein.

a, **c**, **e,** Representative SPR traces of the indicated immobilized proteins in response to the series of concentrations of Jedi1, Jedi2 and Yoda1 measured with affinity mode.

b, **d**, **f**, Dose response curves of mPiezo1 and the L1342A/L1345A mutant proteins to the indicated compounds.

Supplementary Methods

Jedi2 and Yoda1 synthesis. Jedi2 was synthesized by Dr. Wei He's lab. Methyl 2-methylfuran-3-carboxylate (1.5 g, 1 eq.) and N-bromosuccinimide (7 g, 1.1 eq.) were added to dichloromethane (100 mL). The resulting mixture was stirred for 3 h at room temperature. The solution was filtered. Organic layer was washed with water (100 mL x 2) and brine (100 mL x 1) and then dried over anhydrous magnesium sulfate. Filtration to collect the organic layer and concentrated by a rotary evaporator. The crude was purified by column chromatography (PE: EA = 20:1 to 5:1). Intermediate 2 was got as yellow oil (6.1 g, 78.2% yield). Intermediate 2 (4.1 g, 1 eq.), 2 M Na_2CO_3 in water (30 mL), Pd(PPh3)4 (1.1 g, 0.05 eq.) and thiophen-2-ylboronic acid (2.4 g, 1 eq.) were added to toluene/ethanol (100 mL/ 25 mL). The resulting mixture was stirred overnight at 95˚C under Ar atmosphere protected with an Ar balloon. Then the solution was cooled to room temperature and washed with water (50 mL x 2). Organic layer was dried over anhydrous sodium sulfate. Filtration to collect the organic layer and concentrated by a rotary evaporator. The crude was purified by column chromatography (PE: EA = 30:1 to 10:1). Intermediate 3 was got as yellow oil (2.4 g, 57.8% yield). Intermediate 3 (2.0 g, 1 eq.) and sodium hydroxide (1.1 g, 4 eq.) were placed into methanol/H_2O (15 mL/15 mL). The reaction was stirred for 2 h at 70 ˚C. Then methanol was removed by rotary evaporation. The pH of water phase was adjusted to 5-6 with 3N HCl. Filtrate to collect the yellow solid and the solid was washed with water. Yellow solid was dissolved in hexane/ethyl acetate (1:3) and dried over sodium sulfate. The organic layer was passed through a short plug of silica and then concentrated with a rotary evaporator. Jedi2 was got as yellowish solid (1.5 g, 80 % yield). NMR spectrum of the synthesized Jedi2 was the same with the data of Jedi2 from the library.

^{1}H NMR (400M, d6-DMSO, ppm) δ 12.70 (br s, 1H), 7.55 (d, 1H, J = 4.4 Hz), 7.41 (d, 1H, J = 3.2 Hz), 7.10-7.13 (m, 1H), 6.88 (s, 1H), 2.56 (s, 3H). ^{13}C NMR (101M, d6-DMSO, ppm) δ 164.44, 157.58, 146.82, 131.82, 128.20, 125.59, 123.56, 115.75, 105.71, 13.44.

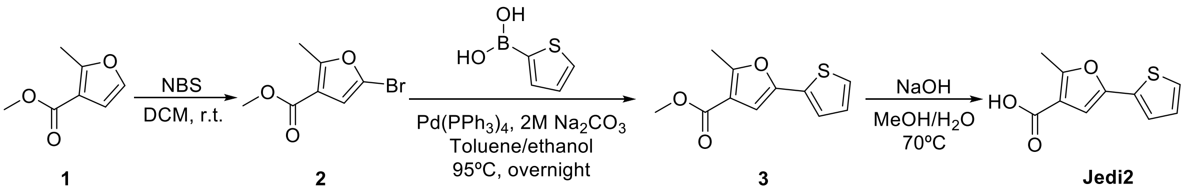

Yoda1 was synthesized by Dr. Liansuo Zu's lab. To a solution of the thiol (392 mg, 2 mmol) in acetone (15 mL) was added K_2CO_3 (304 mg, 2.2 mmol) and 2,6-dichlorobenzyl bromide (526 mg, 2.2 mmol). The mixture was heated to reflux and stirred for 2 h. The reaction was cooled to room temperature and filtered with celite. The acetone was removed under reduced pressure. The residue was recrystallized from ethanol to afford Yoda1 (524 mg, 74%) as white solid.

^{1}H NMR (400 MHz, CDCl3): 9.55(s, 1H), 8.66 (s, 1H), 8.60 (s, 1H), 7.37-7.32 (m, 2H), 7.24-7.18 (m, 1H), 5.01 (s, 2H); ^{13}C NMR (100 MHz, CDCl3): 168.2, 168.0, 145.9, 144.9, 144.4, 142.5, 136.5, 131.8, 130.1, 128.7, 34.6.

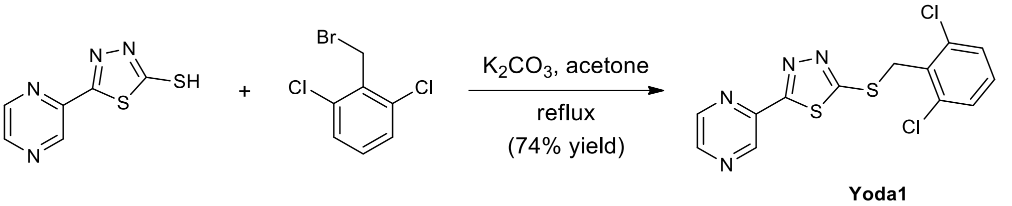

Supplement: Supplementary file 1 — Supplementary Information (PDF 5829 kb)(PDF 5421 kb) [file 41467_2018_3570_MOESM1_ESM.pdf]
